# SCFAs promote intestinal double-negative T cells to regulate the inflammatory response mediated by NLRP3 inflammasome

**DOI:** 10.18632/aging.203487

**Published:** 2021-09-07

**Authors:** Shuiliang Ruan, Liping Zhai, Shasha Wu, Caiqun Zhang, Qiaobing Guan

**Affiliations:** 1 Department of Center Laboratory, The Second Affiliated Hospital of Jiaxing University, Zhejiang, China; 2 Department of Neurology, The Second Affiliated Hospital of Jiaxing University, Zhejiang, China; 3 Department of Pharmacy, The Second Affiliated Hospital of Jiaxing University, Zhejiang, China

**Keywords:** short-chain fatty acids, double-negative T cells, NLRP3, neuroinflammation, Alzheimer’s disease

## Abstract

Short-chain fatty acids (SCFAs) are a product of intestinal bacteria metabolism. Our previous study has found that intestinal bacteria in patients with Alzheimer’s disease (AD) can promote the activation of NLRP3 inflammasome and mediate neuroinflammation. In this study, we mainly explored the regulation of intestinal microenvironmental immunity by intestinal bacterial metabolite SCFAs and the mechanism of NLRP3 activation. First, wild-type (WT) and APP/PS1 mice were intervened with SCFAs. As a result, the proportion of double-negative T cells (CD3^+^CD4^−^CD8^−^, DNTs) in the intestine was increased, SCFAs could promote the expression of intestinal NLRP3 and inflammatory factors (IL-18, IL-6 and TNF-α). Moreover, SCAFs could also promote the level of inflammatory factors in the cerebrospinal fluid (CSF) of mice and aggravate the cognitive impairment in AD mice. CD3^+^ T cells isolated from the spleen were pre-treated with SCFAs, followed by detection of the proportion of DNTs. Consequently, SCFAs could promote the formation of DNTs, activate OX40 signal and simultaneously up-regulate the protein expression of Bcl-2, Bcl-xl and Survivin. Knockdown of OX40 could inhibit SCFAs-induced differentiation of DNTs. The co-culture of DNTs and intestinal macrophages showed that DNTs could activate Fas/FasL-TNF-α signal and induce the activation of NLRP3 inflammasome. In AD mouse models, treatment with Fas and TNFR1 inhibitors could significantly inhibit SCFAs-induced NLRP3 activation and inflammatory factors, while attenuate the inflammatory response in the brain tissue of mice and improve the cognitive ability of mice, however, without significant effect on the level of DNTs.

The present study showed that SCFAs can promote the formation of DNTs through OX40. DNTs could induce the activation of NLRP3 inflammasome and the release of inflammatory factors in macrophages through Fas/FasL-TNF-α signals, thereby increasing the level of inflammatory factors in the central nervous system. When Fas and TNFR1 were inhibited by suppressing the functions of DNTs and macrophages, the activation of NLRP3 was inhibited. DNTs are affected by SCFAs, which is a new mechanism of neuroinflammation in AD.

## INTRODUCTION

Alzheimer’s disease (AD) is a progressive neurodegenerative disease, without clear specific pathogenesis [1]. At present, the hypothesis of amyloid deposition is generally accepted, and the role of neuroinflammation in the pathogenesis of AD has also gradually attracted attention. APP/PS1 is the most commonly used transgenic mouse model, which can be used to study AD and cognitive impairment, and can simulate the pathophysiological state of AD. It is the accepted AD model [2]. The regulation of gut microbiota on AD is a promising novel therapeutic approach for AD [3]. Gut microbiota can regulate the neuroinflammation of AD by affecting intestinal function, neurogenesis and regulating intestinal metabolites. In addition, abnormal composition of intestinal flora can also increase the permeability of the intestinal barrier, activate the immune system and cause systemic inflammation, thereby damaging the blood-brain barrier, aggravating nerve inflammation and nerve damage, ultimately leading to neurodegeneration [4]. Short-chain fatty acids (SCFAs) are one of the main substances of intestinal bacterial metabolites. Existing studies have revealed that SCFAs can induce central neuroinflammation along with the circulatory system. However, the regulation mechanism of SCFAs on intestinal immunity has not yet been reported [5].

Double-negative T cells (CD3^+^CD4^−^CD8^−^, DNTs) have low content among T cells [6–7]. The exact mechanism of DNTs has been rarely reported at present. In ischemic stroke, DNTs have been found to promote brain tissue damage by inducing central inflammatory response [8], however, the mechanism in AD has not been reported. Our team has previously found that the intestinal bacteria of AD patients can activate the intestinal NLRP3 inflammasome, and intestinal inflammatory factors can reach the central system through the circulatory system, causing the activation of microglia [9]. However, the exact activation mechanism of NLRP3 has not been investigated yet. In this study, we mainly explored the role of SCFAs in regulating the activation of intestinal NLRP3 through DNTs and its effect on ad neuroinflammation.

## MATERIALS AND METHODS

### SCFAs promote DNTs formation and NLRP3 expression in APP/PS1 mice

The animal experiment was approved by the ethics committee of Jiaxing University, Animal experiments conform to animal ethics and animal welfare regulations.

Wild-type C57BL/6J mice (WT mice) and APP/PS1 double-transgenic AD mice (AD mice) were selected. Four-month old AD mice showed senile plaque (SP), cognitive impairment and behavior disorder. Mice were purchased from Beijing Biocytogen Co., Ltd (Beijing, China). Mice were divided into WT, AD, WT + SCFAs and AD + SCFAs groups. Mice in the WT and AD groups were conventionally reared (without SCFAs in the feed). And mice in the WT + SCFAs and AD + SCFAs groups were intervened with SCFAs. Sodium acetate, sodium propionate and sodium butyrate were mixed at a mass ratio of 5%, 4.3% and 3.7%. Four groups of mice were raised in the same environment and measured as follows:

### Morris water maze

The Morris water maze and video system were purchased from Feidi Biotechnology Co., Ltd. (Guangzhou, China). Mice were subjected to adaptive training one day before the experiment. In brief, mice entered from the entrance of the water maze and swam freely for 60 s. Mice were allowed to find the platform by themselves and mice were allowed to stand on the platform for 20 s, and mice were later put back into the cage.

Navigation test: The test lasted for 5 days, and the platform was placed in the fourth quadrant. The timer was started when the mouse entered from the entrance. And the time was recorded after the mouse found the platform and boarded the platform. If the mouse cannot find the platform within 60 s, the mouse was guided to the platform and stand for 20 s. between entrance into water and finding the platform was Escape latency (EL).

Space exploration assay: After removing the platform, and the times of crossing the fourth quadrant and retention time on the original platform within 60 s were recorded after entrance.

### Detection of the proportion of DNTs in the spleen and intestines

Mice were fed with SCFAs for 30 days and were sacrificed by carbon dioxide asphyxiation, followed by extraction of spleen and colon tissues. Preparation of cell suspension by homogenization and adjust the cell concentration to 10^7^/ml. Afterwards, cell suspension was incubated with 20 μl of PC5-CD3, FITC-CD4 and PE-CD8 monoclonal antibodies (BD, MA, USA) at room temperature in the dark for 15 min, washed with pre-cooled PBS and centrifuged at 1500rpm. After mixing with 0.5 ml of PBS, samples were subjected to EPICS XLII flow cytometer (Beckman Coulter, CA, USA), followed by calculation of CD3^+^CD4^−^CD8^−^ cell ratio using CellQuest software.

### The expression of NLRP3 and cleaved-Caspase-1 by immunohistochemistry (IHC)

After sacrificing by asphyxiation, the intestinal tissues of mice were fixed with 4% formaldehyde, embedded in paraffin and sliced, baked at 60°C for 2 h, rinsed in xylene and gradient alcohol. The slices were placed in 0.01 mol/L citrate buffer (PH = 6.0) for antigen retrieval at 98°C using microwave for 20 min. The slices were further incubated with 3% hydrogen peroxide at room temperature for 10 min to eliminate endogenous peroxidase, blocked with 2% bovine serum albumin (BSA) at room temperature for 30 min, incubated with anti-NLRP3 and anti-Caspase-1 and monoclonal antibodies (Abcam, MA, USA) (dilution 1:300). After washing with for 3 times, the slices were incubated with proper secondary antibody (Goat anti rabbit IgG H + L) at 37°C for 15 min, incubated with peroxidase-labeled streptomycin (Maxim Biotechnology Company, Fuzhou, China) for 15 min and wash with PBS for 3 times (5 min each). The slices were further visualized with freshly prepared DAB solution (Dako, Glostrup, Denmark), counterstained with hematoxylin and mounted. For negative control, primary antibody was replaced by TBS. The Olympus-DP72 image acquisition system and the Olympus-BX51 upright microscope of the CRi Nauance multispectral imaging system (Cambridge Research and Instrumentation, MA, USA) were used to acquire pictures and for quantitative analysis.

### Enzyme-linked immunosorbent assay (ELISA)

After sacrificing, the peripheral blood of the tail vein from mice was collected and centrifuged to collect the upper serum. The intestinal tissue and brain tissue were ground in liquid nitrogen, added with 1.0 ml of pre-cooled RIPA lysis buffer (Beyotime Biotechnology Co., Ltd., Shanghai, China) on ice for 30 min and centrifuged at 10000g for 15 min. The supernatant was subjected to the protein quantitation bu using BCA kit (Beyotime Biotechnology Co., Ltd., Shanghai, China). After adjusting the protein concentration, the expression of IL-1β, IL-6, and TNF-α in the peripheral blood serum as well as protein from intestinal tissue and brain tissue was measured by ELISA kit (Nanjing Jiancheng Bioengineering Institute, Nanjing, China) according to the manufacturer’s instruction.

### Western blot (WB)

The protein solution extracted in the ELISA was used for WB. The 8–12% SDS-PAGE gel was prepared. The protein supernatant was mixed with 5x loading buffer (up to 20 μl). After boiling for 8 min, the protein sample was subjected to electrophoresis at 80V, further switching to 120V. The sample was subsequently transferred to membrane at 300mA constant current for 0.5–2 h. The membranes were blocked with 5% skimmed milk for 2 h and incubated with proper primary monoclonal antibodies diluted in TBST. The primary antibodies for intestinal tissue included NLRP3, cleaved-Caspase-1, ASC and Pro-Caspase-1 (dilution 1:500, Abcam, MA, USA). The primary antibodies for brain tissue included IBA-1 and GFAP (dilution 1:500, Abcam, MA, USA). After incubation with primary antibody, the membranes were reacted with HRP-labeled goat anti-rabbit secondary antibody (Abcam, MA, USA). After incubation, chemiluminescence was used for visualization, and Image Pro-Plus 6.0 software was used for optical density analysis.

### The effect of Fas and TNFR1 inhibitors on the expression of NLRP3 in intestinal tissue induced by DNTs

In this study, AD mice were used. AD mice were divided into AD, SCFAs, SCFAs + Fas-IN and SCFAs + TNFR1-IN groups. Fas-IN and TNFR1-IN were inhibitors (MCE, Shanghai, China), which were administered by intragastric administration once a day (50 mg/kg). Three groups of mice were fed with SCFAs. After 30 days, the mice were sacrificed by carbon dioxide asphyxiation, followed by extraction of intestinal tissue, spleen, peripheral blood and brain tissue. The following assays were performed accordingly: (1) the proportion of DNTs in mouse intestine and spleen; (2) detection of the expression of NLRP3 and Caspase-1 in intestinal tissue by IHC; (3) the expression of inflammatory factors (IL-1β, IL-6 and TNF-α) in the peripheral blood serum as well as intestinal tissue and brain tissue by ELISA; (4) the protein expression of NLRP3, cleaved-Caspase-1, ASC, Pro-Caspase-1 in intestinal tissue as well as IBA-1 and GFAP in brain tissue by WB.

### The influence and mechanism of SCFAs on the differentiation of DNTs *in vitro*

After sacrificing healthy C57BL/6J mice, the spleen was extracted and ground in liquid nitrogen to prepare cell suspension, followed by isolation of total lymphocytes by using lymphocyte separation solution (Dakewe Biotechnology Co., Ltd., Beijing, China). The 24-well plate was pre-coated with anti-CD3mAb (100 ng/ml) and incubated overnight. Lymphocytes were washed with 2% FBS in PBS, cultured in RPMI-1640 medium (Sigma, MA, USA) containing 20% FBS, IL-2 (50 U/ml) and IL-4 (30 U/ml). The culture medium was changed every three days. Trypan blue was used to detect cell viability. The lymphocytes were divided into control group (Con) and SCFAs group. Cells in the SCFAs group were co-cultured with acetic acid (5 mM), propionic acid (1 mM) and butyric acid (1 mM) for 24 h. Afterwards, cells of the two groups were collected and subjected to the following assays: (1) Detection of the proportion of DNTs by flow cytometry; (2) Detection of secretory protein of DNTs by ELISA; (3) Detection of protein expression by WB.

### The role of OX40 signal in promoting the differentiation of DNTs in SCFAs

The lymphocytes isolated from mouse spleen were divided into Con group, SCFAs group and SCFAs + anti-OX40 group. Lymphocytes were cultured in RPMI 1640 medium containing 20% FBS, IL-2 (50 U/ml) and IL-4 (30 U/ml). Lymphocytes in the anti-OX40 group was pre-treated with OX40 antibody to inhibit OX40 activation (BioVisio, CA, USA). Afterwards, lymphocytes were subjected to the following assays accordingly: (1) detection of the proportion of DNTs by flow cytometry; (2) detection of the protein levels of OX40, Bcl-2, Bcl-xl and Survivin.

### DNTs induce NLRP3 activation in intestinal macrophages *in vitro*

The Rosettesep antibody adsorption method was used to separate DNTs. The mouse intestinal macrophages (Procell Biotechnology Co., Ltd., Wuhan, China) were divided into Con group and DNTs group. Cells in the Con group were the intestinal macrophages cultured separately, and cells in the DNTs group were co-cultured macrophages and DNTs in Transwell chambers. After incubation for 24h, the following assays were performed.

### Detection of the expression of NLRP3 and Caspase-1 by immunofluorescence (IF)

Intestinal macrophages were fixed with 4% formaldehyde at room temperature for 0.5 h, permeabilized with 0.2% Triton X-100 for 5 min, washed with PBS for 3 times, incubated with NLRP3 or Caspase-1 monoclonal antibody at 4°C overnight, wash with PBS twice, incubated with fluorescent secondary antibody and mounted with 95% glycerol, followed by observation under fluorescent microscope.

### Statistical analysis

Measurement data were expressed as mean ± standard deviation (±s). two-way ANOVA was used for comparison between multiple groups, SNK test was utilized for comparison between groups. SPSS 18.0 software was used for statistical analysis. And *P* < 0.05 was considered to be statistical significance.

## RESULTS

### Effect of SCFAs on DNTs and NLRP3 inflammasome activation in AD mice

**Figure 1:**
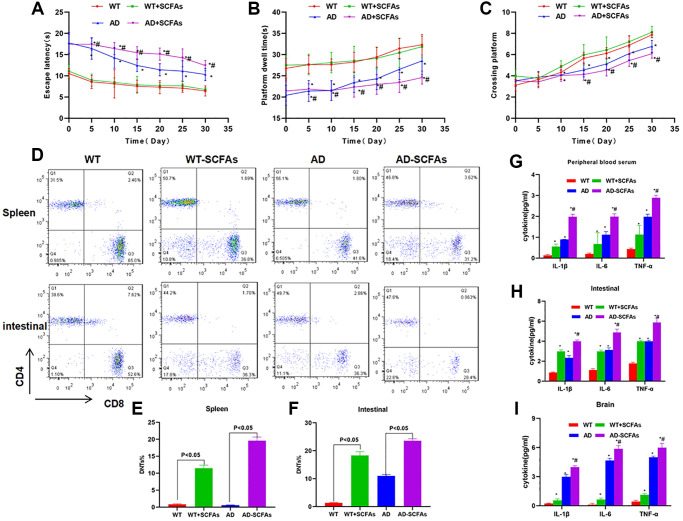
**Effect of SCFAs on the cognitive ability, DNTs differentiation and expression of inflammatory factor in AD mice.** (**A**–**C**) Results of Morris water maze in mice ($\bar{x}\;\pm \text{ s}$, *n* = 10): Compared with WT group at the same time point, ^*^P < 0.05; comparison with AD group at the same time point, ^#^*P* < 0.05. (**D**–**F**) Results of DNTs test ($\bar{x}\;\pm \text{ s}$, *n* = 10) *in vivo*: Comparison of proportion of DNTs in the spleen in (**E**), and comparison of proportion of DNTs in the intestine in (**F**). Statistical significance between the groups, *P* < 0.05. (**G**–**I**) The expression levels of inflammatory factors (IL-1β, IL-6 and TNF-α) in peripheral blood, intestine and brain of mice ($\bar{x}\;\pm \text{ s}$, *n* = 10): Comparison with WT group, ^*^P < 0.05; comparison with AD group, ^#^*P* < 0.05.

**Figure 2:**
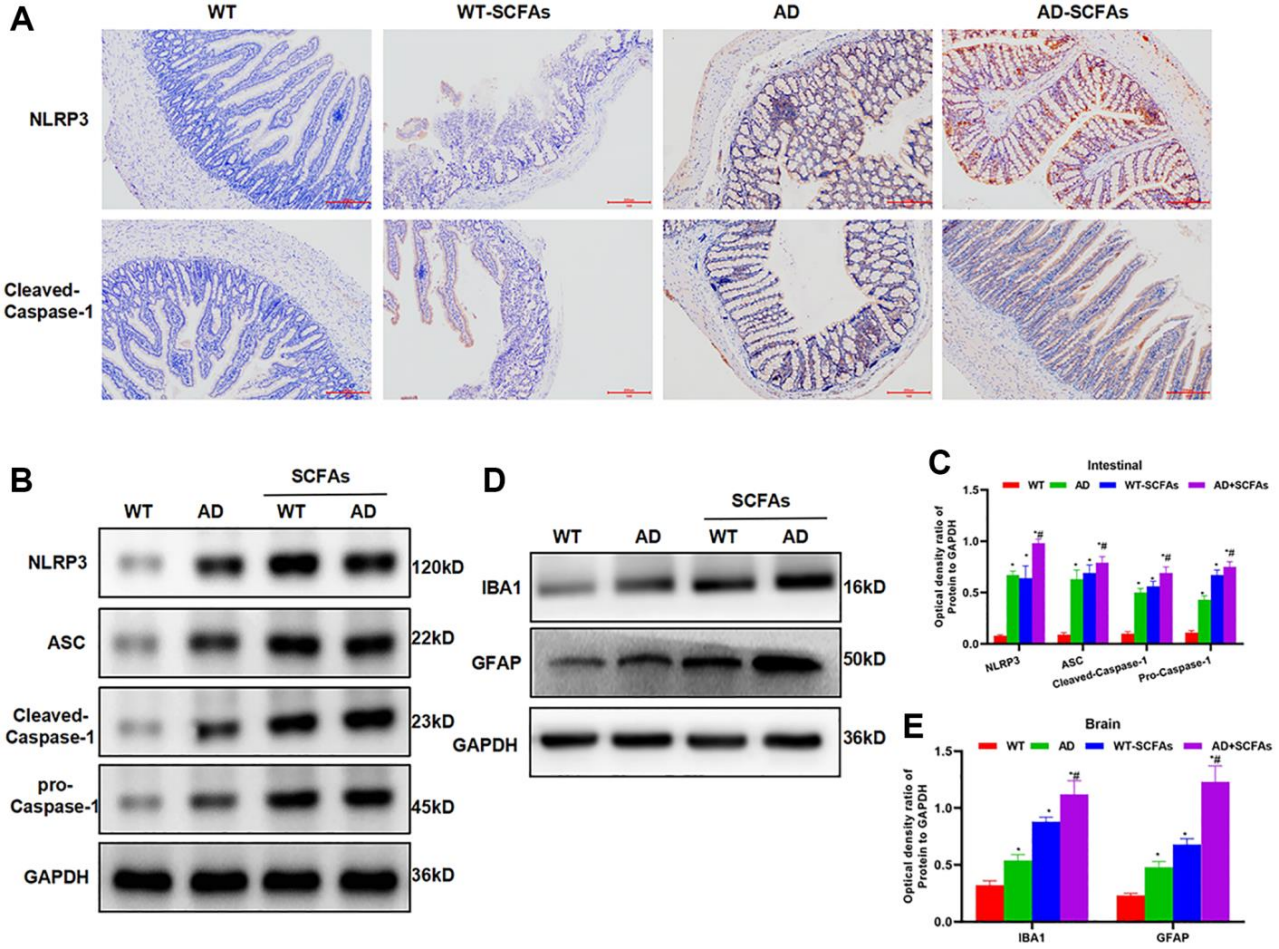
**Effect of SCFAs on the activation of intestinal NLRP3 inflammasome.** (**A**) Detection of mouse intestinal NLRP3 and Caspase-1 expression by IHC (*n* = 5): The expression of NLRP3 and Caspase-1 was negative in the WT group and weakly positive in the WT-SCFAs group. SCFAs promoted the activation of NLRP3. The expression of NLRP3 and Caspase-1 was weakly positive in the AD group, and was positive in the AD-SCFAs group, which was significantly higher than that in the AD group. (**B**–**C**) The expression of NLRP3 inflammasome-related protein in the intestine ($\bar{x}\;\pm \text{ s}$, *n* = 10): The expression of NLRP3, cleaved-Caspase-1, ASC and Pro-Caspase-1 in the NLRP3 inflammasome was low in the WT group. Comparison with the WT group, ^*^P < 0.05; comparison with the AD group, ^#^*P* < 0.05. (**D**–**E**) The protein expression of microglia activation marker (IBA-1 and GFAP) in brain tissue ($\bar{x}\;\pm \text{ s}$, *n* = 10): Comparison with the WT group, ^*^P < 0.05; comparison with the AD group, ^#^*P* < 0.05.

In the behavioral test of mice, we found that the cognitive ability of WT mice was not significantly changed before and after SCFAs intervention. The Morris water maze assay showed that EL, retention time and crossing time were not significantly different between the WT and WT + SCFAs groups. However, the cognitive ability of AD mice was significantly down-regulated after SCFAs intervention, which was significantly different from the AD group (Figure 1A–1C). To detect DNTs, the levels of DNTs in the spleen and intestine of WT and AD mice were relatively low, especially the DNTs in the spleen were not significantly different between WT and AD mice. SCFAs intervention could significantly increase the proportion of DNTs, especially the significantly up-regulated proportion of DNTs in the AD + SCFAs group, which was most remarkable in the intestine (Figure 1D–1F). In terms of inflammatory factors, the expression of inflammatory factors (IL-1β, IL-6 and TNF-α) in the brain tissue, peripheral blood and intestine of the AD group was higher than that in the WT group. After SCFAs intervention, the expression of inflammatory factors in the intestine, brain tissue and peripheral blood of the WT-SCFAs group was significantly higher than that in the WT group, and the expression in the AD-SCFAs group was also significantly higher than the AD group (Figure 1G–1I). IHC showed that NLRP3 and cleaved-Caspase-1 was negatively expressed in the intestine of WT mice and weakly positively expressed in WT + SCFAs, indicating that SCFAs can activate NLRP3 inflammasome. The intestinal expression of NLRP3 and cleaved-Caspase-1 of AD mice was weakly positive, which was positive in AD + SCFAs mice, indicating that SCFAs could promote the high expression of NLRP3 in AD mice (Figure 2A). WB was used to detect the key proteins of NLRP3 inflammasome. As a result, SCFAs could promote the intestinal expression of NLRP3, cleaved-Caspase-1 and ASC. Meanwhile, the expression of microglia activation marker (IBA-1 and GFAP) in brain tissue was also up-regulated, suggesting that SCFAs could activate microglia in brain tissue and promote the release of inflammatory factors (Figure 2B–2E).

### Effect of Fas/Fasl and TNF-α/TNFR1 signal suppression on DNTs and NLRP3 inflammasome

**Figure 3:**
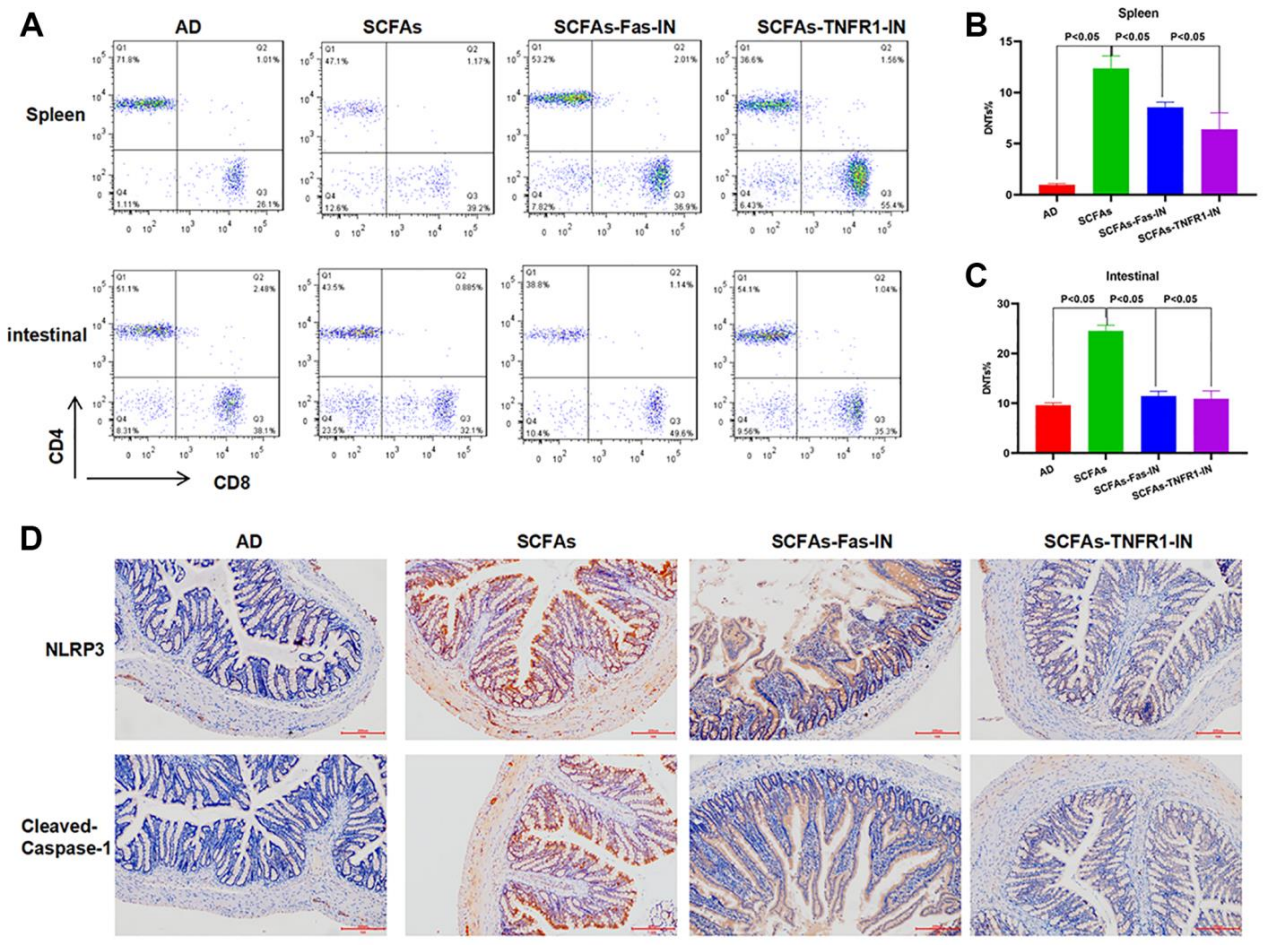
**Effects of Fas-IN and TNFR1-IN on DNTs and NLRP3 inflammasome activation.** (**A**–**C**) Results of DNTs detection ($\bar{x}\;\pm \text{ s}$, *n* = 10): The proportion of DNTs in the spleen of AD mice was relatively low. Comparison of the proportion of DNTs in the spleen in (**B**), and comparison of the proportion of DNTs in the intestine in C. Significant difference between the groups, *P* < 0.05. (**D**) Expression of intestinal NLRP3 and cleaved-Caspase-1 by IHC (*n* = 5): SCFAs could activate the expression of NLRP3 and cleaved-Caspase-1.

**Figure 4:**
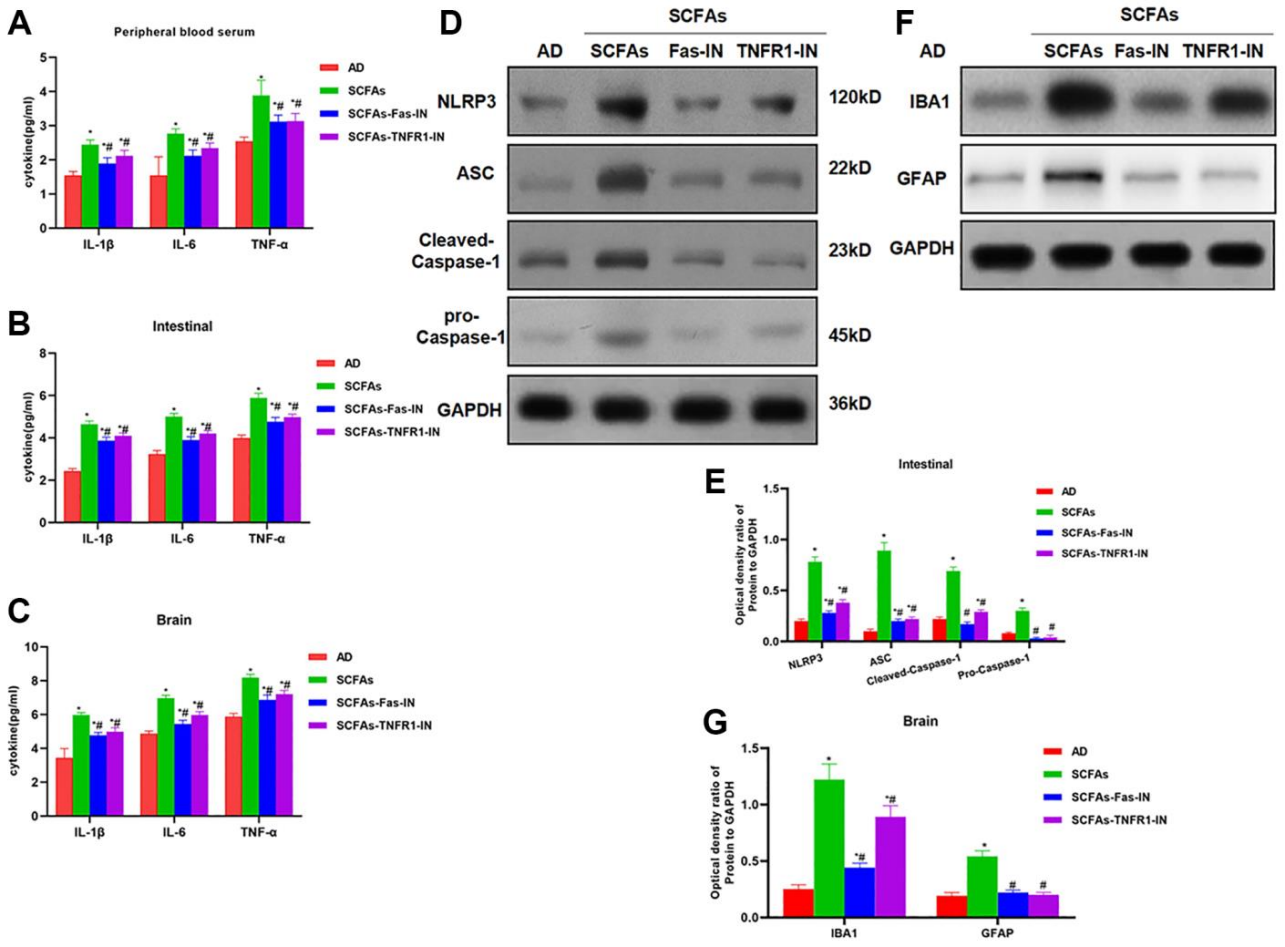
**Effects of Fas-IN and TNFR1-IN on the expression of inflammatory factors and NLRP3 activation.** (**A**–**C**) Results of expression of inflammatory factors ($\bar{x}\;\pm \text{ s}$, *n* = 10): SCFAs could up-regulate the expression of inflammatory factors (IL-1β, IL-6 and TNF-α) in peripheral blood, intestine and brain of mice, comparison with AD group, ^*^P < 0.05; comparison with SCFAs group, ^#^*P* < 0.05. (**D**–**E**) Result of NLRP3 inflammasome activation in mouse intestine ($\bar{x}\;\pm \text{ s}$, *n* = 10): Fas-IN and TNFR1-IN could down-regulate the activation of NLRP3 inflammasome, and down-regulate the expression of NLRP3, ASC, cleaved-Caspase-1 and pro-Caspase-1, comparison with AD group, ^*^P < 0.05; comparison with SCFAs group, ^#^*P* < 0.05. (**F**–**G**) Expression level of microglia activation marker (IBA-1 and GFAP) in brain tissue ($\bar{x}\;\pm \text{ s}$, *n* = 10): Fas-IN and TNFR1-IN could induce the expression of IBA-1 and GFAP. Comparison with AD group, ^*^P < 0.05; comparison with SCFAs group, ^#^*P* < 0.05.

The Fas/Fas1 and TNF-α/TNFR1 signal was blocked to intervene with SCFAs together. As a result, Fas-IN and TNFR1-IN could inhibit SCFAs-induced formation of DNTs. And the proportion of DNTs in the spleen and intestine was down-regulated, which was significantly lower than that in the SCFAs group (Figure 3A–3C). IHC staining showed that Fas-IN and TNFR1-IN could suppress the intestinal expression of NLRP3 and cleaved-Caspase-1. The expression of NLRP3 and cleaved-Caspase-1 in the SCFAs group was significantly higher than that in the AD group, which was weakly positive in AD. While the expression in the SCFAs-Fas-IN and SCFAs-TNFR1-IN group was significantly reduced (Figure 3D). In the detection of inflammatory factors, we also found that Fas-IN and TNFR1-IN could inhibit the levels of inflammatory factors in the brain, intestine and peripheral blood of mice, and resist the SCFAs-induced inflammatory response, which was significantly different from SCFAs (Figure 4A–4C). WB was used to detect the protein expression. The results showed that SCFAs in the intestines induced the expression of NLRP3, which was inhibited by Fas-IN and TNFR1-IN. Besides, the activation of NLRP3 was significantly down-regulated, and the expression of NLRP3, ASC, cleaved-Caspase-1 and pro-Caspase-1 was down-regulated (Figure 4D–4E). The detection of brain tissue showed that Fas-IN and TNFR1-IN could suppress the expression of IBA-1 and GFAP, indicating that Fas/Fasl and TNF-α/TNFR1 signal blocking could inhibit SCFAs-induced microglia activation (Figure 4F–4G).

### SCFAs promoted the formation of DNTs through OX40

**Figure 5:**
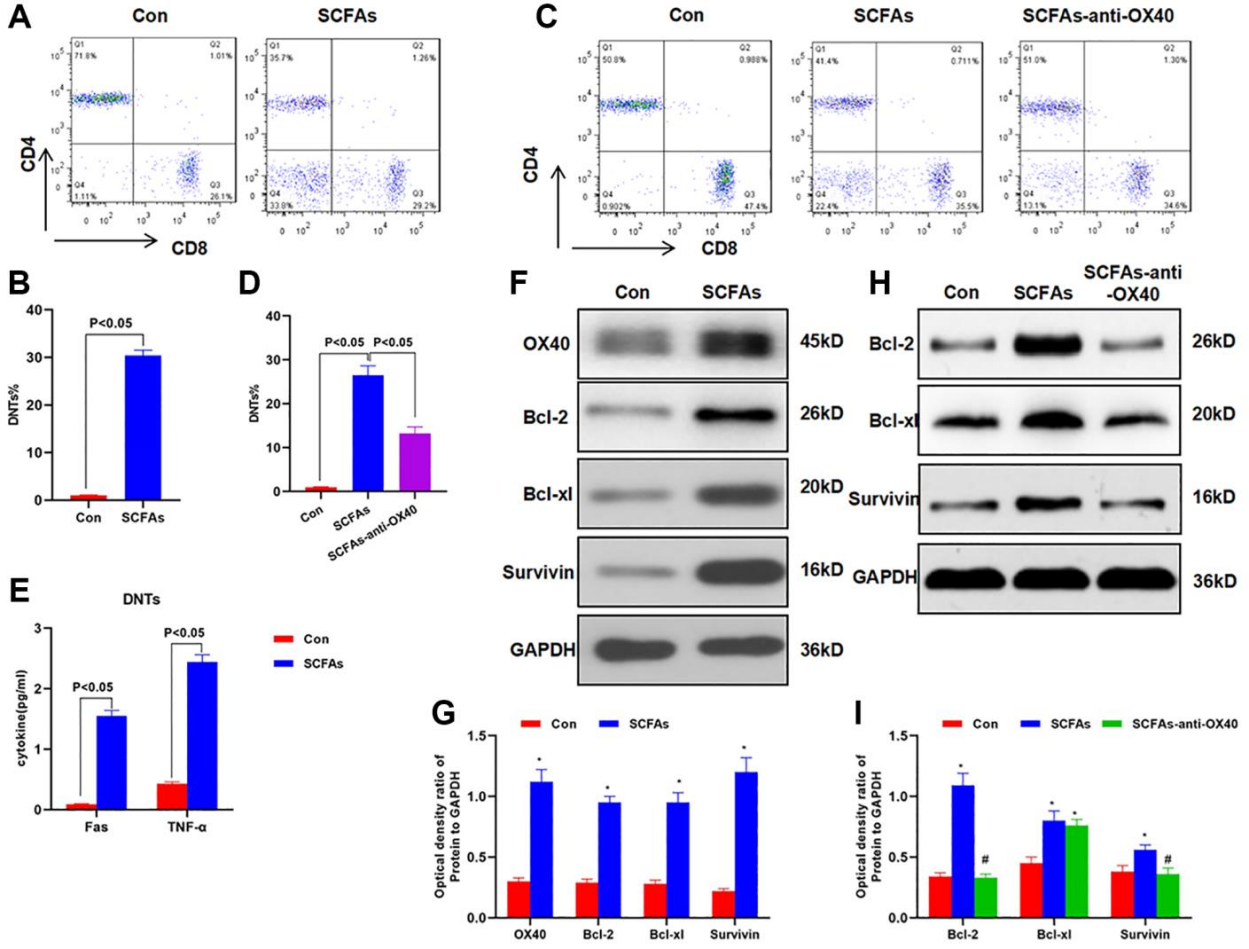
**SCFAs promoted the formation of DNTs through OX40.** (**A**–**B**) SCFAs promoted the formation of DNTs *in vitro* ($\bar{x}\;\pm \text{ s}$, *n* = 3): Cells in the SCFAs group were co-cultured with acetic acid (5 mM), propionic acid (1 mM) and butyric acid (1 mM) for 24 h, The proportion of DNTs in the SCFAs group was increased, and SCFAs promoted the formation of DNTs. Significant difference between groups, *P* < 0.05. (**C**–**D**) Effect of OX40 inhibition on the formation of DNTs ($\bar{x}\;\pm \text{ s}$, *n* = 3): after OX40 inhibition, the proportion of DNTs in the SCFAs-anti-OX40 group was significantly down-regulated than that in the SCFAs group. Significant difference between the groups, *P* < 0.05. (**E**) Detection of Fas and TNF-α secretion in DNTs ($\bar{x}\;\pm \text{ s}$, *n* = 3): the expression of Fas and TNF-α in DNTs was significantly up-regulated, which was significantly higher than that of the Con group. Comparison between groups, *P* < 0.05. (**F**–**G**) Effect of SCFAs on the activation of OX40 in DNTs ($\bar{x}\;\pm \text{ s}$, *n* = 3): the expression of OX40, Bcl-2, Bcl-xl and Survivin was significantly up-regulated in the SCFAs group, which was higher than that of the Con group. Comparison with the Con group, ^*^P < 0.05. (**H**–**I**) Effect of OX40 inhibition on the expression of related protein ($\bar{x}\;\pm \text{ s}$, *n* = 3): anti-OX40 pretreatment could significantly inhibit the expression of Bcl-2, Bcl-xl and Survivin. Comparison with the Con group, ^*^P < 0.05; comparison with the SCFAs group, ^#^*P* < 0.05.

The isolated lymphocytes from the spleen were cultured with SCFAs. As a result, the proportion of DNTs was significantly up-regulated, while the proportion of DNTs without induction was low. When OX40 antibody was pre-treated to block OX40 signal, the proportion of SCFAs-induced DNTs was also down-regulated, which was significantly lower than that of the SCFAs group (Figure 5A–5D). The expression of TNF-α and Fas in DNTs was significantly up-regulated, indicating that DNTs could secrete inflammatory factors (Figure 5E). SCFAs could promote the expression of OX40, Bcl-2, Bcl-xl and Survivin, and promote the survival and proliferation of DNTs cells. OX40 antibody pretreatment could significantly suppress the expression of Bcl-2, Bcl-xl and Survivin (Figure 5F–5I).

### DNTs promoted NLRP3 inflammasome activation and inflammatory response in intestinal macrophages

**Figure 6:**
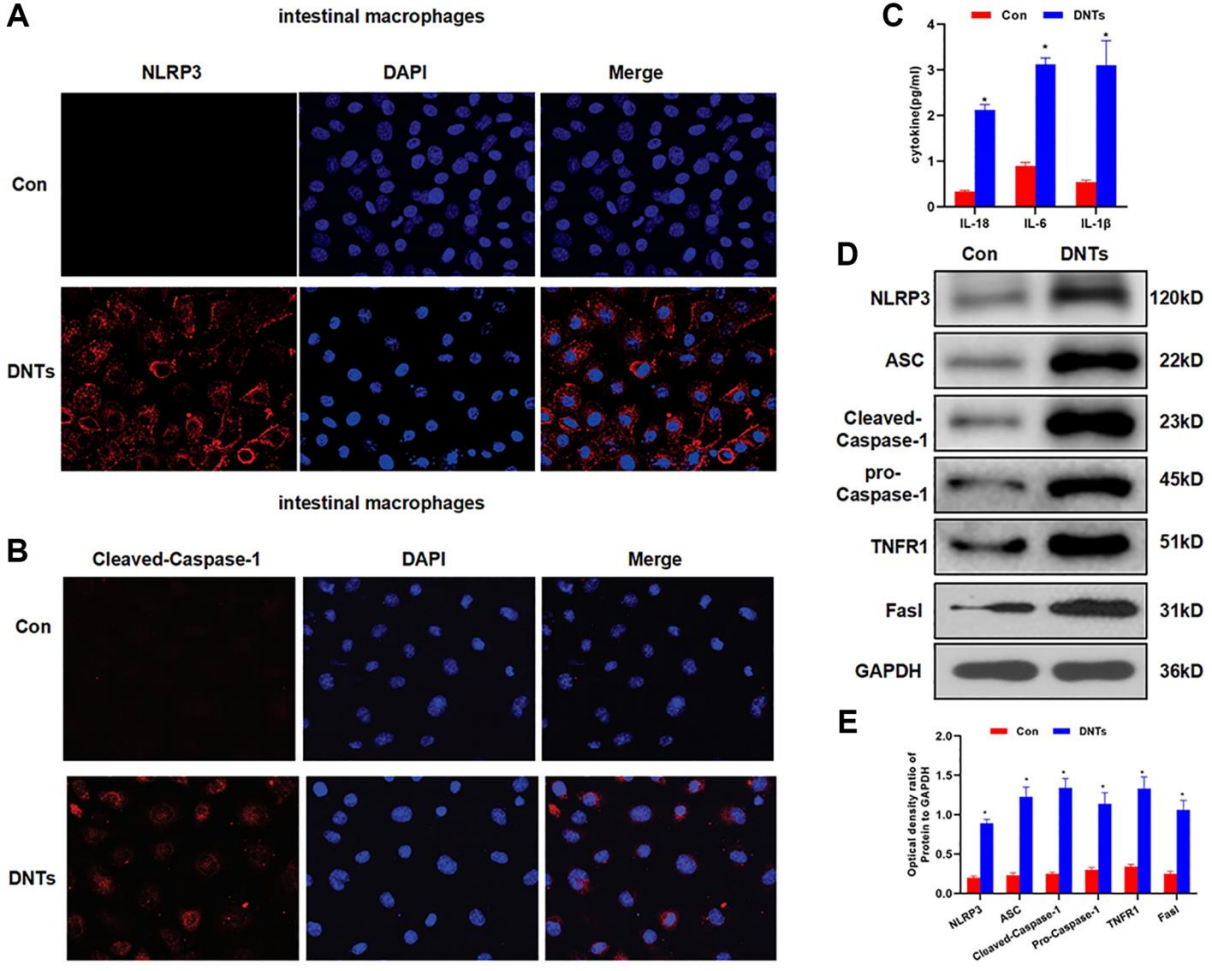
**DNTs promoted NLRP3 inflammasome activation and inflammatory response in intestinal macrophages.** (**A**–**B**) Detection of the expression of NLRP3 and Caspase-1 by IF staining (*n* = 3): NLRP3 and Caspase-1 was not expressed in the Con group, and the expression level was up-regulated after co-culture with DNTs. (**C**) Effect of DNTs on macrophage activation and the expression of inflammatory factors ($\bar{x}\;\pm \text{ s}$, *n* = 3): the level of inflammatory factors in the Con group was low. Comparison with Con group, ^*^P < 0.05. (**D**–**E**) Effect of NLRP3 inflammasome and related protein expression ($\bar{x}\;\pm \text{ s}$, *n* = 3): DNTs co-culture significantly activated the expression of NLRP3 inflammasome related proteins, comparison with the Con group, ^*^P < 0.05.

The isolated DNTs from the spleen were co-cultured with intestinal macrophages. IF staining showed no expression of NLRP3 and cleaved-Caspase-1 in cells from the Con group, while co-culture of DNTs could activate the expression of NLRP3 and cleaved-Caspase-1 in macrophages (Figure 6A–6B). The detection of inflammatory factors showed that the co-culture of DNTs significantly up-regulated the expression of IL-6, IL-18 and IL-1β, which was significantly higher than that of the Con group (Figure 6C). Moreover, we also found that DNTs could promote the expression of NLRP3, cleaved-Caspase-1, pro-Caspase-1 and ASC, which was significantly higher than that of the Con group. Meanwhile, the expression of Fasl and TNFR1 in macrophages was also increased (Figure 6D–6E).

## DISCUSSION

SCFAs are produced by the fermentation of dietary fiber, starch, oligosaccharides, etc., mainly including acetic acid, propionic acid and butyric acid [10]. Existing studies have revealed that SCFAs can regulate blood sugar and regulate bowel function [11–12]. In the regulation of immune function, existing reports have demonstrated that SCFAs produced by intestinal bacteria in patients with multiple sclerosis are associated with the development of the disease [13–14]. In antigen-induced arthritis models, SCFAs could promote the progression of clinical symptoms, which is mainly related to innate immunity regulated by SCFAs [15]. In the study of AD, Harach et al. [16] have found that Firmicutes, Verrucomicrobia, Proteobacteria and Actinomycetes are significantly decreased, while Bacteroides and Tenericutes are significantly increased. Lukens et al. [17] also found in AD model mice that the behavioral changes and brain tissue pathological changes of mice of different ages are closely related to the intestinal bacterial spectrum [18]. The above findings strongly suggest that there may be differences of gut microbiota the between AD patients and healthy people. Moreover, gut microbiota might also play an important role in disease progression and even symptom phenotype. SCFAs can function as metabolites to activate central nervous system microglia and further promote the inflammatory response, suggesting that intestinal bacteria can promote AD progression through the metabolism of SCFAs [8].

DNTs have unique biological functions. The proportion of DNTs in the peripheral blood of normal humans and mice is low, generally around 1–2% [19]. Functional studies have revealed that DNTs can inhibit the functions of CD8^+^ T and CD4^+^ T cells [20]. The study of neurological diseases has found that DNTs lay a pro-inflammatory effect by promoting the activation of microglia in mice with ischemic stroke, which is associated with the secretion of TNF-α [8]. However, the role in AD has not been reported. Our study has found that the proportion of DNTs in AD mice is relatively high, and SCFAs can induce the differentiation of DNTs. When the proportion of DNTs is increased in the intestine, NLRP3 inflammasome is activated. We find that SCFAs-DNTs-NLRP3 may be a novel signal of AD-related inflammation. Our team has previously found that the intestinal bacteria in AD patients could activate the intestinal NLRP3 inflammasome [9], which could further release inflammatory factors to reach the central system through the circulation to induce central inflammation. In this study, we found that SCFAs also activated the activation of NLRP3, and SCFAs are precisely one of the important pathogenic metabolites of gut microbiota in AD. When DNTs are activated and massively formed, the inflammatory factors in the peripheral blood, intestinal tract and central system are up-regulated, and DNTs are immune cells that can release TNF-α [21]. Therefore, Fas and TNFR1 inhibitors were simultaneously applied with SCFAs intervention. The results showed that Fas-IN and TNFR1-IN could resist the effects of SCFAs, the proportion of DNTs was down-regulated, and the expression of NLRP3 was also down-regulated. NLRP3 inflammasome is a complex protein that can be induced by various inflammatory factors. TNF-α and Fas are the two inflammatory factors. TNF-α and Fas can mediate the formation of NLRP3-Caspase-1-ASC through the membrane receptor protein, further promoting the cleavage of pro-IL-1β to form mature IL-1β, thereby playing a role as inflammatory factors after secretion [22]. The increased proportion of DNTs could activate the microglia in the brain tissue, and up-regulated the expression of IBA-1 and GFAP, which is consistent with our previous results [23–24]. To further explore the role of DNTs, we isolated spleen lymphocytes of mice and found that the proportion of spleen lymphocytes DNTs was relatively low. SCFAs intervention could increase the proportion of DNTs. It is reported that OX40 is a regulatory signal for the formation of DNTs [25–26]. We have also found that SCFAs can promote the expression of OX40, and simultaneously promote the proliferation of DNTs by up-regulating Bcl-2 and other protein. The above functions of SCFAs were suppressed after OX40 inhibition, therefore, we speculate that the role of SCFAs and OX40 signal is related. Finally, DNTs and mouse intestinal macrophages were co-cultured. The results showed that DNTs could induce the activation of NLRP3 inflammasome in macrophages, activate Fas1 and TNFR1, and up-regulate the levels of inflammatory factors. The results indicate that DNTs definitely activate NLRP3 inflammasome, mediate the release of inflammatory factors, and ultimately inflammatory factors aggravate the neuroinflammatory response of AD through the circulation.

However, some studies have found that some kinds of SCFAs play a regulatory role in amyloid deposition [27]. Some studies have also found that esterified SCFAs can exert central anti-inflammatory effect through GPR4 [28], SCFAs also play an important role in the regulation of microglia function, [29]. However, a considerable number of studies have found that SCFAs can promote the deposition of amyloid protein and the activation of microglia [30]. So, we can say that SCFAs is a double-edged sword, which plays an important role in the occurrence and development of AD. In the central system, SCFAs may play a role through microglia and amyloid, but this role is related to the type, concentration and time of SCFAs. Our research focuses on the role of SCFAs in intestinal immune regulation, and we find that SCFAs promotes DNTs differentiation. Therefore, our research results are quite different from the previous SCFAs related research results. We did not study the effect of SCFAs on the central nervous system, but started with intestinal immunity to reveal the role of brain gut axis. This has a certain innovative significance.

## CONCLUSIONS

In our study, we found that DNTs are a new type of immune cells that can promote the progression of ad neuroinflammation by activating intestinal NLRP3. SCFAs can promote DNTs differentiation through OX40. These findings are the extension of our previous results, further revealing the roles of SCFAs in AD. Hopefully, the gut microbiota metabolism and immune regulation mechanisms could be combined, and DNTs are a new type of promising immune cells for AD research.
